# Scar Tissue after a Cesarean Section—The Management of Different Complications in Pregnant Women

**DOI:** 10.3390/ijerph182211998

**Published:** 2021-11-15

**Authors:** Aleksandra Stupak, Adrianna Kondracka, Agnieszka Fronczek, Anna Kwaśniewska

**Affiliations:** 1 Department of Obstetrics and Pathology of Pregnancy, Independent Public Teaching Hospital No 1 in Lublin, Medical University of Lublin, 20-081 Lublin, Poland; adriannakondracka@wp.pl (A.K.); anna.kwasniewska@umlub.pl (A.K.); 2 Department of Pathomorphology, Independent Public Teaching Hospital No 1 in Lublin, Medical University of Lublin, 20-081 Lublin, Poland; a.m.fronczek@gmail.com

**Keywords:** cesarean scar pregnancy, cesarean section, pregnancy, cicatrix, niche, ultrasound scan, management

## Abstract

The definition of a cesarean scar pregnancy (CSP) is the localization of the gestational sac (GS) in the cicatrix tissue, which is created in the front wall of the uterus after a previous cesarean section (CS). The worldwide prevalence of CSP has been growing rapidly. However, there are no general recommendations regarding prophylaxis and treatment of the abnormalities of the anterior wall of the uterus discovered in a non-pregnant myometrium, or how to deal with existing cases of CSP. We present the latest knowledge, a holistic approach to the biology, histology, imaging, and management concerning post-CS scars based on our cases, which were treated in the Department of Pregnancy and Pathology of Pregnancy in the Medical University of Lublin, Poland. In our study, we present images of tissue samples of areas with a cicatrix in the uterus, and ultrasound and MRI images of CSP. We discuss the advances in the biology of the post-CS scar tissue, the prevention techniques used to repair the scar defect (niche) before the pregnancy, and the treatment of different complications of CSP, such as the rupture of the gravid uterus or the dehiscence of the myometrium.

## 1. Introduction

The number of performed cesarean sections is continuously raising. In the United States, the average of CSs done in 2007 was over 30% [1,2]. In 2010, in China, it reached up to 60%, while in the private sector in Brazil it was near to 80% [1,2]. In Poland, the prevalence of CS is about 30% but is growing rapidly [3].

The increasing frequency of CS being performed is mainly due to a planned, repeat cesarean (an indication of the threatened rupture of the uterus); electronic fetal monitoring as a gold standard; and the decreasing number of spontaneous vaginal breech deliveries. Although, there are the possibilities of a successful vaginal delivery in women with a cesarean section history in 60–80% of cases [4]. Vaginal birth after a cesarean section (VBAC) is now an integral part of modern obstetrics.

A previous CS increases the threat of pathological placentation [5]. Therefore, advances in obstetrics and the biology of the wound after a hysterotomy are of interest for all obstetricians.

A hysterotomy cicatrix is defined as a hypo- or hyper-echoic line in the anterior lower wall of the uterus resulting from a prior cesarean delivery [6]. A CS scar is therefore made up of two components: an apparent defect, and the scar tissue joined with the myometrium.

The wound healing is a complex process, which takes place in three stages: the inflammation of the serum of the damaged blood vessels (in the first days—homeostasis and immune system reactivity), proliferation (up to 4 weeks—granulation and neovascularization), and maturation or remodeling (up to 1–2 years—collagen formation, deposition, and remodeling) [7]. For the proper restoration of the wound, these processes must occur in the correct sequence and timeframe, because the final effect is the reconstruction of the incision area. It is suggested that the same processes and timelines take place in the repairing of a cesarean hysterotomy incision [8]. The latest progress in the research of cesarean scars shows the involvement of transforming growth factor beta (TGF-b), connective tissue growth factor (CTGF), basic fibroblast growth factor (bFGF), platelet-derived growth factor (PDGF), vascular endothelial growth factor (VEGF), and tumor necrosis factor alfa (TNF-a) in the scarring process. Nevertheless, there are still too few studies concerning the pathologies of CS scars, both in physiological and in pathological conditions.

The inadequate healing of the uterus after a cesarean section has potential long-term consequences, including the thinning of the muscle layer, which happens in up to 60% of cases [9]. This defect is associated with obstetrical and gynecological complications, such as: ectopic scar pregnancies, placenta accreta spectrum (PAS), uterine rupture, intracycle spotting, dysmenorrhea, pelvic pain, and infertility.

Anomalies in a cesarean scar, initially described as ‘‘isthmocele’’ by Morris, and also termed “niche” by some authors, can be visualized by hysterosalpingography, transvaginal sonography, saline infusion sonohysterography, hysteroscopy, and MRI, and are illustrated by a defect within the myometrium [9,10]. For the illustration of the cicatrix tissue on the uterus, ultrasound waves or magnetic resonance is used. In an ultrasound scan, the scar in the lower uterine segment (LUS) can vary from normal-appearing, and practically homogenous from an unscarred one, to paper-slim with a poor visualization of the uterine muscle layer contents [6]. Sometimes, a scarred LUS is interrupted, which leads to uterine dehiscence (the subperitoneal partition of the uterine scar, with the chorioamniotic membrane noticeable through the peritoneum) or, rarely during contraction, uterine rupture. The danger of uterine rupture in the attendance of a previous niche cannot be predicted. The thinning of the lower uterine segment is the effect of the extending caused by the gestation itself, which does not arise in other scarred tissues [4]. Cicatrix tissue is rigid and will not stretch. During labor, the descent of the fetal head may stretch and eventually rupture the wall of the uterus.

The latest evidence suggests that the risk of a niche relates to the number of previous cesarean sections or the mode of uterine suture used. After one CS, the defect rate can reach 61%, but after three, it is almost 100% [11]. However, there is no compromise on the method of uterine closure following a cesarean delivery in using one or two layers of stiches, the locking or not of the first layer, and whether the decidua should be in- or excluded [9,12].

One of the rarest but most dangerous complications in pregnancies after a previous CS is the nidation of embryo in the area of the scar tissue. CSP is one the rarest type of ectopic pregnancies [13]. According to the latest overview by NICE Guidelines, the rate of ectopic pregnancy is 11 per 1000 pregnancies [14]. The presence of scar tissue in pregnancy is an obstetrical nightmare for the lack of its effective treatment. Its prevalence has been reported as 1/2200–1/1800 pregnancies, and is increasing because of the higher number of CSs being performed [15]. The diagnosis of CSP is a difficult task and requires experience. The primary diagnostic tool is a vaginal ultrasound. The criteria for the early identification of CSP in the first trimester are:

1 an empty uterine cavity with clear endometrium and empty endocervical canal,
2 the detection of a gestational sac within the anterior lower segment of the uterus embedded in the cesarean scar,
3 an absent or thin (<5 mm) myometrium layer between the gestational sac and the bladder,
4 a peritrophoblastic color Doppler flow around the sac with low-impedance (pulsatility <1), high-velocity flow (<20 cm/s), a resistive index of less than 0.5, and a peak systolic/diastolic flow ratio of <3 [16,17],
5 the pathologies of the adnexa should be excluded, and there should be no detection of fluid in the Douglas pouch unless in the case of a massive hemorrhage or rupture of the uterus [5].

The latest advances in the biology, histology, and imaging of the mark are allowing professionals to avoid and treat more effectively the defects of the scarred LUS.

### 1.1. Case 1

We present the case of a 33-year-old woman, admitted to our Department, with a diagnosis of a CSP with a previous complicated obstetrical history (G4P1A2). Her first pregnancy ended with a CS without complications in 2007 due to threatened fetal distress at 39 weeks’ gestation (wks). In 2011, she had a spontaneous abortion with dilatation and curettage caused by the remnants of a miscarriage. In 2017, there was another spontaneous abortion at 6 wks without surgical treatment.

**Figure 1:**
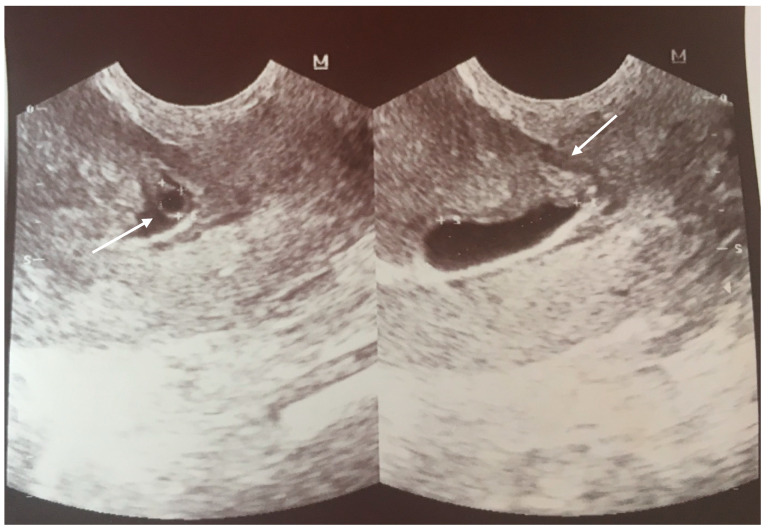
Transvaginal scan at 6 wks with a visible gestational sac in the area of the scar after CS (the arrows are pointing to GS and scar).

The first checkup of the current, fourth, pregnancy taken in the Out-Patients Clinic was performed at 6 wks gestation. The US scan revealed the gestational sac in the area of the scar from the previous cesarean section, and a single embryo with heart activity (Figure 1).

This was the first time of the diagnosis. However, no management was offered to the patient, ipso facto, and it was confirmed to her that the pregnancy was developing physiologically normally.

**Figure 2:**
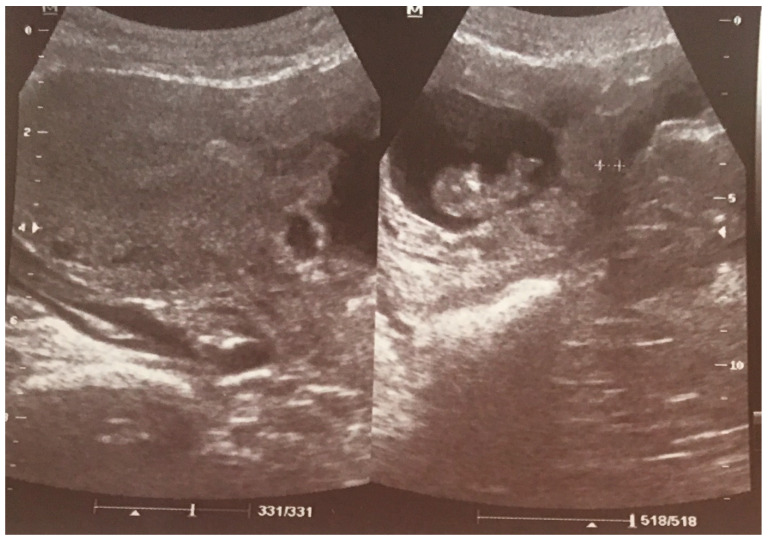
US scan at 10 wks with measurement of the thickness of the scar about 5.4 mm.

At 10 wks, following another scan, the trophoblast was covering the scar from the CS, and the thickness of the scar was measured at 5.4 mm (Figure 2).

At 13 wks, the patient was hospitalized in a state hospital due to bleeding from the genital tract. She was discharged without any scan and with a recommendation of iron supplementation.

At 14 wks gestation, she was again hospitalized in the same hospital because of cramps in the lower abdominal area. The trophoblast was covering the cesarean scar with possible ingrowth into the urine bladder wall and uterine muscle; the scar thickness was estimated at 2.5 mm in this region. After pharmacological treatment for pain, the patient was dismissed at her own decision.

**Figure 3:**
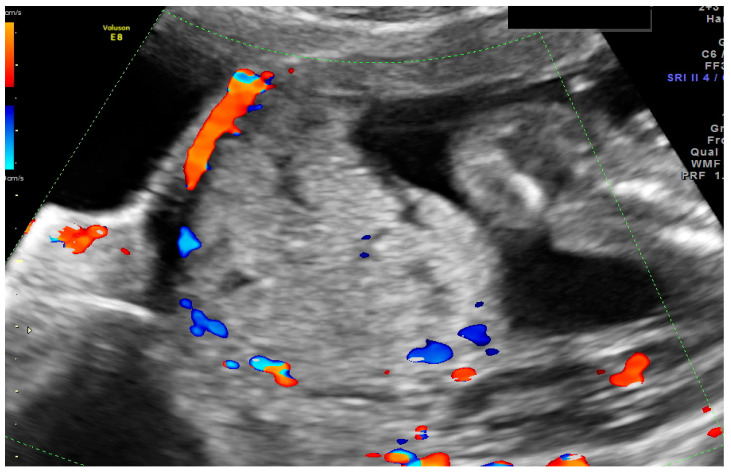
The transabdominal scan at 15 wks of the lower uterus segment with massive vascularization between the placenta and urine bladder.

At admission to our Department at 15 wks, the patient reported lower abdominal pains and bleeding. During hospitalization, a diagnosis of CSP was finally confirmed, and explained to the patient with a possible life-threating condition (Figure 3).

**Figure 4:**
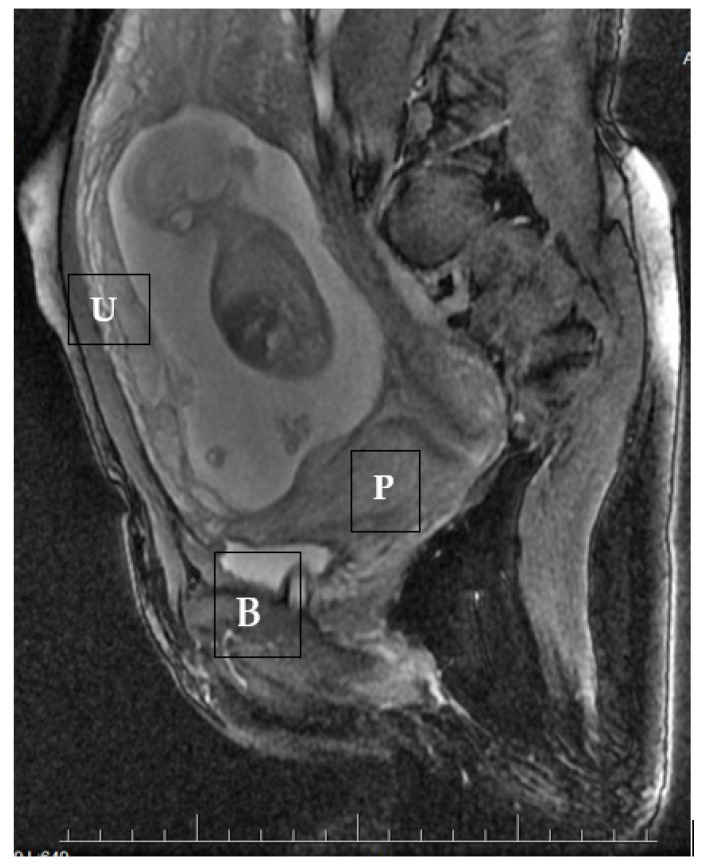
A magnetic resonance sagittal image of the cesarean scar pregnancy at 15 weeks, showing the infiltration of the trophoblast into the uterine wall and towards the bladder. U—uterus, P—placenta, B—bladder.

During ultrasound scanning, we found the placenta covering the cesarean scar, with a very high risk of ingrowth into the uterine muscle and urine bladder. In the cystoscopic and MRI images, there was no evidence of placental growth into the bladder (Figure 4).

**Figure 5:**
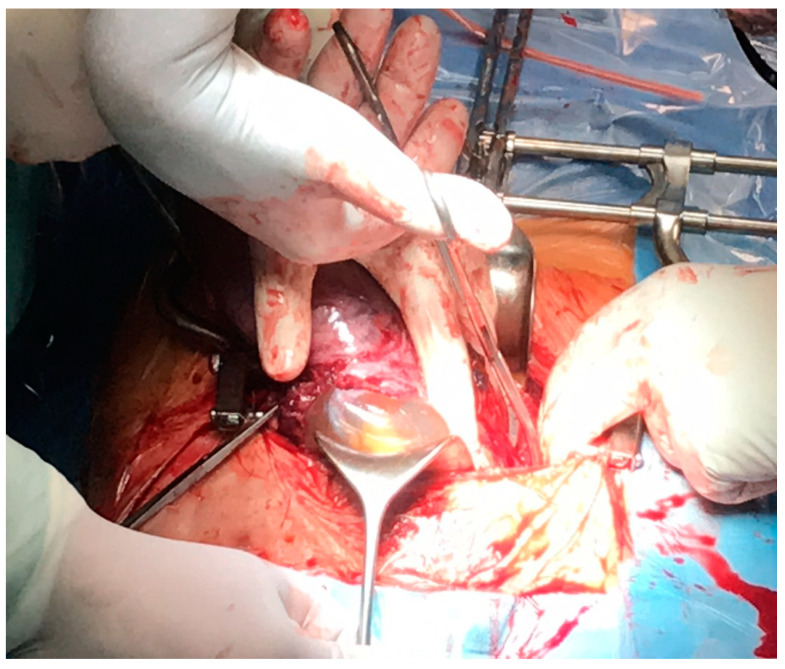
Intraoperative image after the separation of the peritoneum from the anterior wall of the uterus.

At 17 wks, in the Department, there was suddenly extensive bleeding from the genital tract and severe pain in the abdomen. The patient was informed about the life-threatening state and was qualified for embolization of the uterine arteries in the Department of Radiology and an urgent hysterectomy. The uterine embolization procedure was performed under a local anesthetic of the right femoral artery via the Seldinger method (embolization balls and shredded sponge). During the operation, we discovered a total rupture of the front wall of the uterus and a damaged wall of the urine bladder (invasion of the trophoblast villi and crack in the all layers of the bladder wall) (Figure 5).

**Figure 6:**
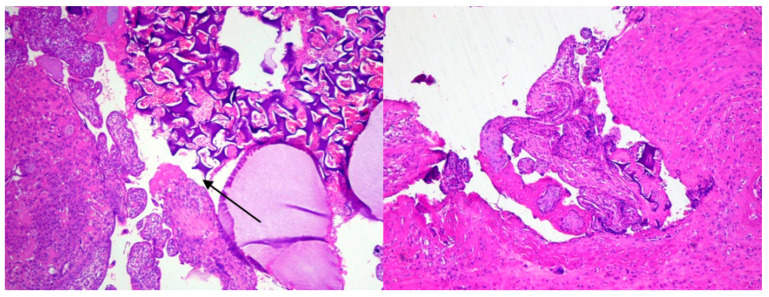
The placental villi, fragment of decidua, and foreign body. (H&E stain, 4×). The uterus wall from the area of the scar after caesarian section. Chorionic villi attach to the muscular layer of the uterus wall (absent decidua basali) and fragment of foreign body (arrow). (H&E stain, 4×).

A typical abdominal hysterectomy was performed. Despite the embolization, the patient lost 1500 mL of blood. After the blood transfusion, her condition was stable. The histological staining of the scar tissue taken from the uterus is presented below (Figure 6).

In the scar area, pieces of soluble threads after previous CS were found.

### 1.2. Case 2

A 35-year-old patient at Gravida 2, Para 1 was admitted to our Department at 21 wks because of an ache in the lower abdomen for 2 days. The previous CS was performed at a different hospital and we had no information about the used operation technique at 40 wks, as a consequence of the threatened asphyxia of the fetus, in 2010.

**Figure 7:**
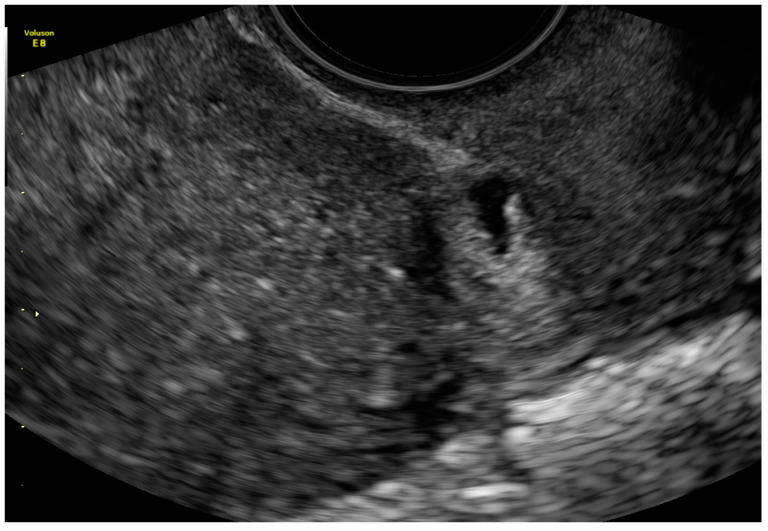
The sagittal section of uterus by vaginal probe with visible “niche” of cesarean scar area in size 3 × 7 mm.

The first scan she had after the CS was when she missed menstruation. During this scan, an hypoechogenic irregular area was found in the mark from the CS (Figure 7).

Confirmation of an intrauterine pregnancy could not be made at that time, so the patient was informed of the existence of a “niche” and advised as to the laparoscopic repair of the scar tissue.

**Figure 8:**
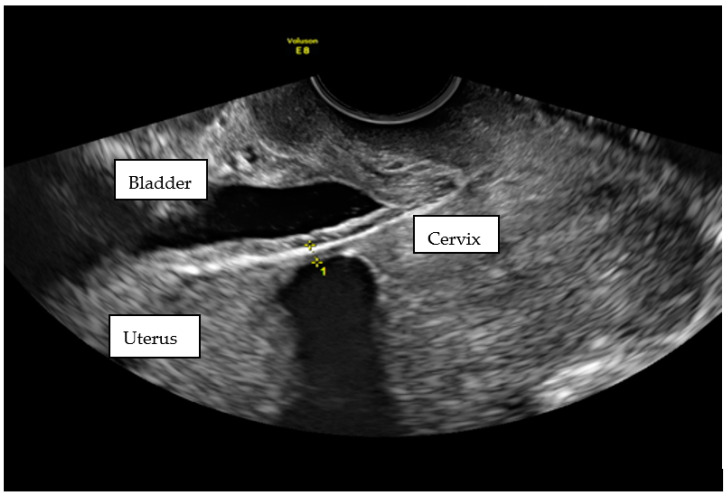
Transvaginal scan of pregnant lower segment of uterus at 21 wks. The measurement of the scar thickness was 3.4 mm.

Unfortunately, in the next scan, a pregnancy at 6 wks was confirmed. No “niche” was mentioned. The patient had a First Trimester Screening Program for chromosomal abnormalities at 12 wks, performed by a Fetal Medicine Foundation licensed obstetrician. No abnormalities were found. When she came for the second scan in this Program, she complained about cramps. In the scan, the LUS had thinned to 3.4 mm with dehiscence of the myometrium (Figure 8).

**Figure 9:**
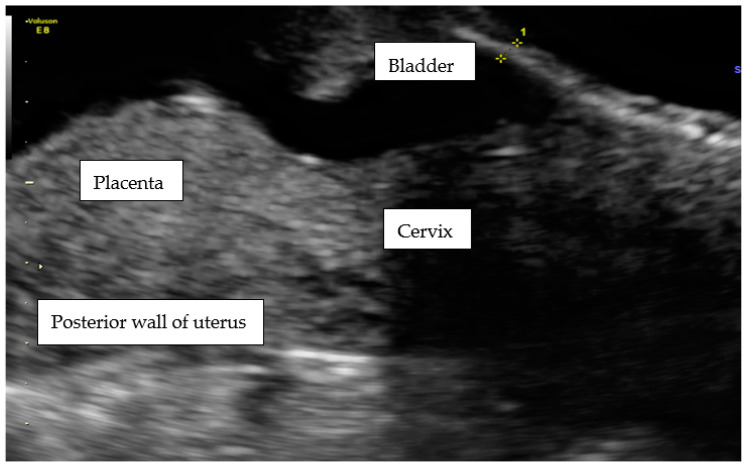
The transabdominal scan at 25 wks with measurement of scar thickness of 2.6 mm.

The patient was admitted to our Department for observation and spent 5 weeks there (from 21 wks to 25 wks). After prophylactic steroid treatment for lung maturation, the patient departed the hospital on her own request. The dismissal scan showed an even thinner scar (Figure 9).

At 36 weeks of gestation, the patient underwent a scheduled cesarean section, which was performed 7 years after previous surgery. The area of the scar (2.6 mm) was only covered by a thin layer of peritonea with a total dehiscence of the myometrium. The placenta was on the posterior wall of the corpus of the uterus. The uterus was sutured typically (double-layer continuous), and the patient came through the postoperative period well.

## 2. Discussion

We have presented two cases that illustrate the most widespread complications of cesarean scar areas in the LUS. In the first case, the scar tissue was complicated by the presence of the gestational sac. Diagnoses of CSP are growing nowadays, perhaps due to the rising number of CSs being performed. The progression in the visualization of an early pregnancy via US is an improvement in fast and proper management. It is proved that the US scan is the most valuable, repeatable, and cost-effective technique [18,19]. Scar niche can be assessed by a combined integrated 2D and 3D US scan with new specific geometrical and anatomical considerations [20].

In our first patient, most of the inclusion criteria for CSP mentioned above were obtained during the scan at 6 wks, so the diagnosis was made correctly [5]. In the opinion of many authors, in the second or third trimester of pregnancy, cases of CSP are almost indistinguishable from an ingrown placenta [21,22]. Therefore, it is crucial to perform the first scan with particular discernment [23]. The woman should be given every kind of information about the type of pathology in the uterine scar, so she can make the appropriate decision regarding medical treatment.

Another method of imaging cesarean scar abnormalities is magnetic resonance [7]. In our case, all the requirements for the diagnosis of CSP were met, so the use of MRI was to evaluate the deepness of the placenta protrusion into the bladder. The same method, with the same effect, was used in the second case [24].

Nevertheless, no treatment was advised to the patient with the CSP at the time of diagnosis. In our last publication in 2014 concerning the topic, we had a similar case [5]. A diagnosis of CSP was suggested at 6 wks, but the pregnant woman did not agree to invasive treatment for personal reasons. However, after the confirmation of lethal abnormalities in the fetus, we performed an embolization and hysterectomy at 13 wks. Other cases were managed expectantly, evolved into placenta accreta/increta, and led to severe maternal morbidity and hysterectomy because of uterine rupture [25]. It might seem that a CSP is a precursor of placenta accreta/percreta [18]. Therefore, the majority of the cases diagnosed early are terminated surgically or pharmacologically. In this case, the expectant management led to severe complications in the 2nd trimester of the pregnancy [26,27].

In first case, the CSP was complicated by massive bleeding after the rupture of the uterus and a life-saving hysterectomy. However, there are many examples of the conservative management of this uterine scar tissue abnormality. Conservative treatment includes systemic and local methotrexate, uterine artery embolization, the use of local embryocides such as potassium chloride, and sac aspiration, as well as combinations thereof [12,13,28].

The managing of CSP depends individually on the capability of the obstetrical care center [23].

1. Expectant management only after detailed explanation about risks (rupture, bleeding palcenta percreta)
2. Conservative methods like medical treatment, or/and mechanical interventions depending on what the facilities can provide
3. The decision for the procedure is influenced by the available management and expertise of the center when complications occur.

Our facility is a tertiary care unit with full access to consultations with other experts, such as pathologists, urologists, radiologists, surgeons, anesthesiologists, and neonatologists. The emergency management was detailed interdisciplinarily, and there was no chance of leaving the uterus after the rupture of the front wall and massive bleeding. There is still no algorithm for CSP, and most of the literature published is based on case reports or small number of patients.

It is believed that CS does not increase the risk of miscarriage in future pregnancies [29], although it has been reported that CS niche may lead to infertility or spontaneous miscarriages if the implantation is close to or in the niche [30,31]. Moreover, several cases of CSP misdiagnosed as spontaneous miscarriages (bleeding) have also been reported [32]. It cannot be ruled out that the previous two times of spontaneous abortion were CSP-related. CSP is not a physiological process of implantation, so probably miscarriage is “a natural” solution.

In the latest literature, the histological analysis in 78.9% of cases, taken in a survey after the laparoscopic repair of the cicatrix tissue, revealed such pathological findings as the incidence of fibrotic tissue [1]. In the residual 21.1% cases, the scar had signs of endometriosis, defined as the presence of endometrial glands inside the scar unconnected to the endometrial surface on serial sections. Nezhat et al. also observed coexistent endometriosis at the site of the niche [33]. In our first example, in the histological staining, the pathological findings revealed a very thin myometrium layer, placenta accreta in the area of the scar post CS, and fragments of a foreign body (probably a chemoembolization material). Because of the decidualization of the endometrium, no signs of endometriosis were found. The absence of decidual basali is in keeping with the findings of other authors who performed histopathological reports [24]. In our first case, the threads from previous CS were found which means that the patient might have had healing problems. The wound healing concept says the scar needs 1–2 years but the sutures are soluble even earlier. No other histopathological findings were detected.

Uterine dehiscence is related to the modified biochemical behavior of the scarring process [8]. The myometrium in the scarred LUS showed a higher collagen content, an increase in the concentration of VEGF, FGF, and TNF-α, but a reduction in TGF-ß and CTGF. Those several factors that are involved in the scarring process could be considered as some biomarkers for the diagnosis of CS niche. This management might be cost-effective for the prevention of CSP or in the follow-up after previous CS. We encourage more studies. In cases of rapid postpartum involution, the LUS with hysterotomy scar might be displayed to greater tension and subsequent disruption with a “life-threatening state” [7].

Vikhareva Osser et al. made the interesting suggestion that the disturbance of the wound healing process after a cesarean might be connected with the performing of a CS in advanced labor [34]. Another study was performed by Pomorski et al. on the measurements of the uterine scar, which were taken from 409 women with a history of at least one low transverse CS with a single layer uterine closure [10]. The mean residual myometrial thickness (RMT) value was significantly smaller in women with the CS performed in the second stage of labor compared with women without a cervical dilatation and with women in the first stage of labor. Moreover, the decrease in RMT significantly correlated with the number of CSs. In both of our patients, the CSs were performed after many hours of regular contraction and the engaged dilatation of the cervix, which might be not consistent with those findings.

The appearance of CS scar defects in an ultrasound scan may be clinically relevant, but there is limited evidence relating the scar’s appearance to the uterine function in a future pregnancy. The distinction between a complete uterine wall rupture and uterine scar dehiscence is important. The latter is not associated with a major risk for either the fetus or the mother, while the former poses a major risk for both [35].

The 2nd patient had uterine scar dehiscence, in our opinion a defect found in early pregnancy, but which had developed fully by the time of the growth of the uterus and physiological contractions. There is no evidence about what time during the gestation period scar dehiscence appears in US scanning. Our case is not the only one that combined the existence of the niche in non-pregnant/early gestation pregnancy with a second trimester scar dehiscence [36].

The obstetrical complication of a prior defect is unknown while a large niche could be related to uterine rupture [34]. In our study, the thickness of the scar tissue when the patient left the hospital was 2.6 mm, which can be preserved as “normal”. Since, in many studies, the cut-off value for the sudden rupture of uterus was 2 mm, the question is, what is the best time to assess the thickness of the scar tissue in pregnant women? In the work cited, the authors performed the US transabdominal scan 2 weeks before delivery. The sonographic measurements were then correlated with the visual findings of a uterine scar at the time of the cesarean section. Regarding our case, is it safe to prolong the pregnancy at 25 wks because the scar is 3.4 mm? The management of an incomplete myometrial and serous dehiscence has never been recorded [36]. Should elective CS be performed in order to prevent the emergency rupture of the uterus? Unfortunately, there are no sufficient biological or clinical data to allow us to develop algorithms capable of predicting intrapartum outcomes with an acceptable level of precision [7]. In one case, a patient with a diagnosed niche before pregnancy was admitted to hospital at 30 wks because of repeated and painful contractions [36]. A US scan during the contractions showed the protrusion of the amniotic membranes (like a hernia) through the complete dehiscence of the uterine scar. An emergency CS was preformed due to the threatened complete rupture of the uterus. The authors emphasis that measuring the changes of the thickness of the LUS during pregnancy has been associated with fluctuating results, and it does not enable the prediction of a preterm rupture or to avoid prematurity.

The pathology of LUS in the first patient was detected 10 years, and in the second patient 7 years, after the CS, which is consistent with other authors’ findings, in that the passing of time has no impact on the occurrence of a scar defect. The earliest measurement of scar tissue in LUS was made 6 wks after the CS [10,37]. In the study by Dosdela et al. there were no dissimilarities in the thickness of this area, even after 6 months.

The repair treatment, proposed by the obstetrician who performed the first scan, would be the most appropriate for a future pregnancy. Other authors have also confirmed this procedure as being successful and cost-effective [1]. Cesarean scar defects have been described for over 20 years, and laparoscopic repair has been performed for over 15 years [38]. Lately, there are three methods for the surgical excision of a cesarean scar defect: hysteroscopic resection, laparoscopic resection and repair, or repair of the niche through a vaginal approach. The laparoscopy method is optimal for patients who want fertility due to improved visualization and the capability to resuture the myometrium using a two-layer closure. Hysteroscopic resection may also participate a role in certain cases, for its safety, cost-effectiveness, and quick recovery time. The cut-off criterion to include a patient for the proper type of management might be the width of the scar area. For a scar under 3 mm, a laparoscopic approach should be proposed, because of the higher risk of urine bladder damage [33]. In the previously cited work by Donnez et al., a series of 38 symptomatic women with cesarean scar defects and a remaining myometrial thickness of less than 3 mm, according to MRI, had a laparoscopic repair of the defect [1]. The mean thickness of the myometrium improved significantly from 1.43 ± 0.7 mm before surgery to 9.62 ± 1.8 mm after surgery, and 44% of the patients became pregnant after the procedure.

We possess no knowledge about the technique of the closure of the uterus in the CS in both patients. The newest and most widespread method of performing a CS is a transverse lower cut on the muscle of the uterus, but the suture technique is different in different care centers. In the literature, there are attempts to compare double-layer sutures with single-layer sutures in the course of wound healing and scar development after the CS and the origination of LUS defects. In a 3-arm 1:1:1 randomized study in women with singleton pregnancies undergoing elective primary CS at 38 wks’ gestation performed by Roberge et al., a total number of 81 patients were enrolled for the analysis [9]. The closure of the uterine scar was carried out by a locked single-layer including the decidua, a double-layer with locked first-layer including the decidua, or a double-layer with unlocked first-layer excluding the decidua. The latter technique was related to a greater remaining myometrium thickness, total myometrium thickness, and healing ratio, suggesting that it is associated with better healing of the uterine scar. Furthermore, in other authors’ opinions, this method may possibly lead to a reduction in severe obstetric complications regarding scar tissue [39,40,41].

## 3. Conclusions

Taking into consideration the growing advances in scar biology, the CS scar must be evaluated via the use of a standardized method recommended for evaluating the CS scar to identify any potential risk factors that may affect its healing, and the development of the niche and severe obstetric complications like CSP, PAS, or uterus rupture.

## 4. Clinical Implication

We would strongly recommend for a routine US check-up in a previous CS patient. We encourage a higher index of suspicion for CSPs during an early pregnancy ultrasound. Moreover, the index of CS scar tissue/niche should be highlighted and monitored through US scanning. All obstetric/gynecologic practitioners performing US scans need continuous training and standardized diagnostic and management protocols. We support an international CSP registry at www.csp-registry.com (accessed on 15 November 2018).

## Data Availability

Not applicable.

## Abbreviations

CS	cesarean section
CSP	cesarean scar pregnancy
CTGF	connective tissue growth factor
GS	gestational sac
FGF	fibroblast growth factor
LUS	lower uterine segment
PAS	placenta accrete spectrum
RMT	residual myometrial thickness
wks	weeks’ gestation
VBAC	vaginal birth after a cesarean section
VEGF	vascular endothelial growth factor
TNF-α	tumor necrosis factor α
TGF-ß	transforming growth factor β
